# Voice Assistants as Consultants for Male Patients with Sexual Dysfunction: A Reliable Option?

**DOI:** 10.3390/ijerph20032612

**Published:** 2023-02-01

**Authors:** Luigi Napolitano, Biagio Barone, Lorenzo Spirito, Francesco Trama, Savio Domenico Pandolfo, Marco Capece, Esther García-Rojo, Esaú Fernández-Pascual, Felice Crocetto, Ferdinando Fusco, Marco De Sio, Davide Arcaniolo, Celeste Manfredi

**Affiliations:** 1 Unit of Urology, Department of Neurosciences, Reproductive Sciences, and Odontostomatology, University of Naples “Federico II”, 80131 Naples, Italy; 2 Unit of Urology, Department of Woman, Child and General and Specialized Surgery, University of Campania “Luigi Vanvitelli”, 80131 Naples, Italy; 3 Urology Unit, “Santa Maria Delle Grazie” Hospital, 80078 Pozzuoli, Italy; 4 Department of Urology, Hospital Universitario HM Sanchinarro, 28050 Madrid, Spain; 5 LYX Institute of Urology, Faculty of Medicine, Universidad Francisco de Vitoria, 28006 Madrid, Spain

**Keywords:** Alexa, Google Assistant, Siri, sexual health, voice assistant

## Abstract

The aim of this study was to evaluate the ability of Google Assistant, Alexa, and Siri to recognize and answer questions about male sexual health. Each VA was tested on a smart speaker: Alexa on Amazon Echo Dot 4th Gen., Google Assistant on Google Home Mini, and Siri on Apple HomePod. A pool of patients’ frequently asked questions regarding erectile dysfunction (ED), premature ejaculation (PE), Peyronie’s disease (PD), male infertility, and other aspects of male sexual health were identified by authors. The recognition of question was evaluated (“yes” or “not”). For each recognized question, the response characteristics (domains) were rated on a scale from 0 to 10 (according to the quality). We chose the recognition rate of the questions as the primary outcome and the quality of the answers as the secondary outcome. Overall, the best VA in recognizing questions was Siri, with a total of 83.3% questions compared with 64.0% for Alexa (*p* = 0.024) and 74.0% for Google Assistant (*p* = 0.061). Siri was associated with a significantly higher recognition rate than Alexa for PE (80% vs. 40%; *p* = 0.002) and PD (66.7% vs. 33.3%; *p* = 0.010). The quality of the responses was classified as low in 57 out of 105 cases (54.3%), intermediate in 46 cases (43.8%), and high in only 2 cases (1.9%), highlighting an overall intermediate-low quality of the answers. Male infertility was the condition associated with the highest mean scores in “Targeted response to the problem” (7.32 ± 2.57), “Scientific correctness of the answer”, (5.9 ± 2.76) “Completeness of the answer” (5.14 ± 2.56), and “Understandability of the response for a patient” (5.3 ± 2.51) domains. Siri was associated with significantly higher scores than Alexa (*p* < 0.05) in several domains of all conditions evaluated. The question recognition rate of VAs is quite high; however, the quality of the answers is still intermediate-low. Siri seems superior to Alexa in both question recognition and response quality. Male infertility appears to be the sexual dysfunction best addressed by VAs.

## 1. Introduction

Nowadays, the Internet has become an integral part of most people’s lives. People use the Internet via personal computer (PC) or smartphone for all kind of research and often to find health information [1]. There are several reasons why patients seek answers to their health problems through the use of the Internet. First of all, the easy and immediate access to data means that it is much easier to type a few keywords into a search engine than go to a physician. Moreover, the web search is free and there is no interlocutor who can judge or cause shame. Patients generally search the web before meeting a doctor to find out if they actually need professional help with their condition, while searching is carried out after the medical visit if the patients are dissatisfied or doubtful with the information received [2,3]. The Internet can be a useful tool to find a doctor and in some cases to solve simple doubts or give comfort to the patient; however, it can lead to misinformation and encourage self-diagnosis and self-prescription of ineffective, inappropriate, or even harmful treatments. Indeed, the Internet contains a large amount of inaccurate or incorrect medical information because anyone can write anything without control, rules, or guidelines. Furthermore, despite the presence of websites with scientifically correct information, patients generally do not know how to reach them or simply do not have the tools to correctly interpret what they read [4].

Voice assistants (VAs) are software agents that can recognize spoken questions and respond via synthesized voices. They are designed to respond in natural language, simulating human conversation. VAs use the Internet to provide answers; however, they cannot be considered as simple “web search engines 2.0” since they are artificial intelligence (AI) [5,6]. VAs are integrated into smartphones, smart speakers, laptops, and desktops to help users in a variety of daily tasks or for entertainment purposes. Currently, the most popular VAs are Amazon Alexa, Apple Siri, and Google Assistant; in fact, they are incorporated in most smartphones and smart speakers on the market. In particular, smart speakers with Alexa are the best sellers, while Google Assistant and Siri are the most popular VAs on smartphones. Microsoft Cortana and Samsung Bixby are other examples of VAs that are much less used [7]. It has been estimated that 142 million Americans (42.1% of the population) used a VA in 2022; most of them were between the ages of 19 and 54. Furthermore, a progressive increase in their use is estimated in the coming years [8]. It is reasonable to state that VAs are used by people to search for health information; however, papers evaluating their reliability for this purpose are very limited [9,10]. The Internet is used by patients to search for information about sexual dysfunction, including erectile dysfunction (ED), male infertility, premature ejaculation (PE), and Peyronie’s disease (PD), as well as by young men to inquire about sexual health [11,12]. To date, there is no article available in the literature investigating the use of VAs to search for information on sexual health.

The aim of this study was to evaluate the ability of Google Assistant, Alexa, and Siri to recognize and answer questions about male sexual health.

## 2. Materials and Methods

### 2.1. Device Selection

We tested each VA on a smart speaker, selecting the best-selling devices at the time of the study [13]: Alexa on Amazon Echo Dot 4th Gen. (Amazon.com Inc., Seattle, WA, USA), Google Assistant on Google Home Mini (Google Inc., Mountain View, CA, USA), and Siri on Apple HomePod mini (Apple Inc., Cupertino, CA, USA). All devices were owned by the authors, in perfect functional condition, set for the English language, and updated to the latest software available at the time of the investigation. All devices were tested in the same quiet place with a stable internet connection in Naples (Italy).

### 2.2. Identification of Question Pool

The authors identified a pool of possible patients’ frequently asked questions (FAQs) regarding ED, PE, PD, male infertility, and other aspects of male sexual health. FAQs were chosen on the basis of a consensus among the authors, with a senior author (DA) in charge of solving any discrepancies. All authors had proven experience in the management of andrological patients. In the end, 75 questions were identified, 15 for each condition (ED, PE, PD, male infertility, other) (Table S1). All questions were grammatically reviewed by a native English speaker. Each selected question was formulated by 2 native English speakers (1:1 sex ratio) to all devices to rule out the tone of voice possibly affecting the answers. All native speakers answered the question “How much experience do you have using VAs?” to have at least good experience.

### 2.3. Collection of Answers, Data Interpretation, and Outcomes

The answers were collected in October 2022. All questions from native speakers and responses from VAs were recorded with an audio recorder. The recognition of question was evaluated (“yes” or “not”). The questions were deemed recognized whenever the VA formulated an answer, regardless of its characteristics. Absence of answer, affirmation of not having understood, and affirmation of inability to help were classified as unrecognized questions. For each recognized question, the following response characteristics (domains) were subjectively rated on a scale from 0 to 10 (according to the quality): the targeted response to the problem, the scientific correctness of the answer, the completeness of the answer, the understandability of the response for a patient, the use of empathic language, and, only when mentioned, the authoritativeness of the scientific source cited and the referral to other appropriate sources of help. Arbitrarily, a mean domain score of 0–3 was associated with low quality, >3 but <7 with intermediate quality, and 7–10 with high quality. The evaluation of all answers was performed by two authors (LN, BB) independently; for each characteristic, the mean of the two assigned scores was considered. The maximum difference in score tolerated for each evaluation between the two authors was 3 points; for larger differences, a third senior author (DA) assigned the final score. We chose the recognition rate of the questions as the primary outcome and the quality of the answers as the secondary outcome.

### 2.4. Statistics

The categorical variables were described as frequencies and percentages, while the quantitative variables were presented as mean and standard deviation (SD). The Chi-Square test and the ANOVA test with Bonferroni post hoc analysis were used to analyze the data. The *p*-value threshold was arbitrarily set at 0.05. All statistical analyses were performed using IBM SPSS Statistics^®^ (IBM Corp. Released 2017. IBM SPSS Statistics for Windows, Version 25.0. Armonk, NY, USA: IBM Corp.).

## 3. Results

### 3.1. Recognition of Questions

Overall, the best VA in recognizing questions was Siri, with a total of 83.3% questions compared with 64.0% for Alexa (*p* = 0.024) and 74.0% for Google Assistant (*p* = 0.061). All VAs showed perfectly overlapping recognition rates of ED-related questions (93.3%; *p* = 1.0). A similar recognition rate between VAs (*p* > 0.05) was also found for male infertility (>90% for all VAs) and other aspects of male sexual health (range: 60.0–76.7%). Conversely, Siri was associated with a significantly higher recognition rate than Alexa for PE (80% vs. 40%; *p* = 0.002) and PD (66.7% vs. 33.3%; *p* = 0.010); however, no statistically significant difference was found when comparing Siri and Google Assistant for PE and PD (*p* = 0.091 and *p* = 0.190, respectively). The recognition rates of each VA are detailed in Table 1.

### 3.2. Quality and Characteristics of the Answers

**Figure 1:**
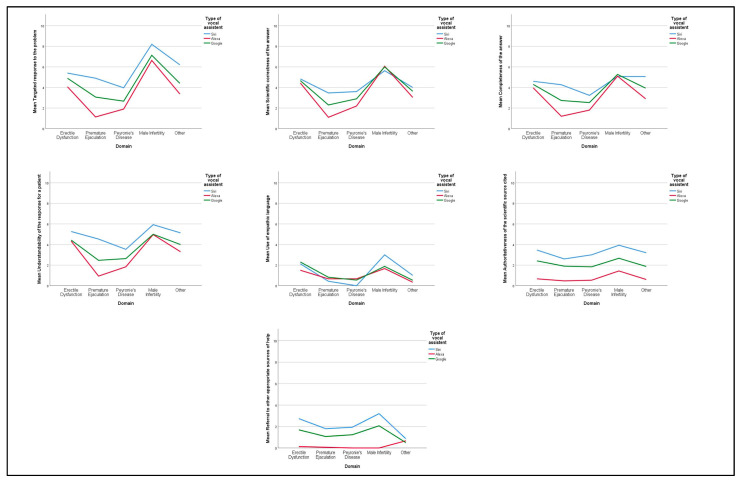
Mean scores of VAs according to the response domains. VA: Voice Assistant.

The quality of the responses was classified as low in 57 out of 105 cases (54.3%), intermediate in 46 cases (43.8%), and high in only 2 cases (1.9%), highlighting an overall intermediate-low quality of the answers. Male infertility was the condition associated with the highest mean scores in “Targeted response to the problem” (7.32 ± 2.57), “Scientific correctness of the answer”, (5.9 ± 2.76) “Completeness of the answer” (5.14 ± 2.56), and “Understandability of the response for a patient” (5.3 ± 2.51) domains. “Use of empathic language”, “Authoritativeness of the scientific source cited”, and “Referral to other appropriate sources of help” were the domains associated with the lower mean scores for all conditions; more specifically, a mean score greater than 3 was attributed only in 3 out of 45 cases (6.7%) (Figure 1).

Considering ED, no statistically significant difference among the VAs was found in the investigated domains except for “Authoritativeness of the scientific source cited” (*p* = 0.001) and “Referral to other appropriate sources of help” (*p* < 0.0001). In particular, a statistically significant superiority of Siri compared with Alexa was reported for the “Authoritativeness of the scientific source cited” (*p* = 0.001), while both Siri and Google were statistically significantly superior to Alexa in terms of “Referral to other appropriate sources of help” (*p* < 0.0001 and *p* = 0.03, respectively). 

Regarding PE, statistically significant differences were reported in all the evaluated domains except for “Use of empathic language” (*p* = 0.791). In particular, Siri had higher scores compared with Alexa in “Targeted response to the problem” (*p* < 0.0001), “Scientific correctness of the answer” (*p* = 0.003), “Completeness of the answer” (*p* = 0.001), “Understandability of the response for a patient” (*p* < 0.0001), “Authoritativeness of the scientific source cited” (*p* = 0.001), and “Referral to other appropriate sources of help” (*p* < 0.0001). When the domains “Referral to other appropriate sources of help” and “Authoritativeness of the scientific source cited” were considered, Google Assistant was also associated with a statistically significant higher score compared with Alexa (*p* = 0.035 in both cases).

In the PD setting, only the domains “Authoritativeness of the scientific source cited” (*p* = 0.001) and “Referral to other appropriate sources of help” (*p* = 0.004) showed a statistically significant difference. More specifically, Siri scored significantly higher than Alexa in these two domains (*p* = 0.001 and *p* = 0.003, respectively). 

Similarly, regarding male infertility, statistically significant differences were reported only for the domains “Authoritativeness of the scientific source cited” (*p* < 0.0001) and “Referral to other appropriate sources of help” (*p* < 0.0001). In particular, Siri scored significantly higher than Alexa (*p* < 0.0001 in both domains) and Google Assistant (*p* = 0.048 and *p* = 0.003, respectively in the two domains).

Finally, taking into account the other aspects of male sexual health, a statistically significant difference was found for the domains “Targeted response to the problem” (*p* = 0.020) and “Authoritativeness of the scientific source cited” (*p* < 0.0001). Siri was associated with a significantly higher score than Alexa in these two domains (*p* = 0.017 and *p* < 0.0001, respectively).

The quality and characteristics of the answers formulated by the VAs are detailed in Table S2. The post hoc analysis is reported in Table S3.

## 4. Discussion

### 4.1. Our Findings and Available Literature

ED [14,15,16,17,18,19,20], PE [21], PD [22,23,24,25,26,27,28,29,30], male infertility [31,32], genital aesthetic concerns [33,34,35,36,37], as well as several other conditions [38,39,40,41,42,43,44,45] can significantly impact the sexual health of male patients. When this happens, worries and doubts often lead patients to independently search for information on the Internet with every available tool [1,2]. VAs have recently joined the arsenal of devices that allow searching for information online [5,6] and, even if there are no studies yet, they are probably already widely used to obtain information on sexual health.

We found that the highest recognition rate overall was associated with Siri (83%), although a statistically significant difference was reported only against Alexa (*p* = 0.024). All VAs showed similar (*p* > 0.05) recognition rates for ED, male infertility, and other aspects of male sexual health. Conversely, Siri was associated with a significantly higher recognition rate than Alexa for PE (*p* = 0.002) and PD (*p* = 0.010). It is important to underline that Google Assistant did not differ significantly (*p* > 0.05) from either Siri or Alexa in the recognition rate of questions regarding all investigated conditions. Even when the individual domains of each condition were considered, Siri was generally associated with statistically superior scores to Alexa, while Google Assistant was associated with often intermediate results between Siri and Alexa. These findings suggest the superiority of Siri over Alexa, while no clear difference emerges between Google Assistant and other VAs. The above differences could be explained by the different technology and algorithms underlying the selection of online information sources.

We reported an overall intermediate-low quality of the answers (98.1%). Therefore, while the questions were often recognized, the reliability of the answers was severely limited. Male infertility was the condition best addressed by the VAs. Conversely, PE and PD were often associated with the lowest scores across domains regardless of VA. This could be explained by the fact that male infertility is often perceived as a health problem more than PD or PE [31,32]; therefore, the greater amount of information online or the higher frequency of questions about this condition may have led to better responses from the VAs. The poor reliability of the answers regarding PE, despite being one of the most frequent male sexual dysfunctions [21], underlines just how much this condition is underestimated and less known by the population.

“Use of empathic language” was among the domains associated with the lower scores for all investigated conditions regardless of VA. This shows that in addition to the content, the form of the responses provided by the VAs also needs significant improvement. The same health information reported in an empathic way can take on a completely different meaning for a worried patient. Of course, we are aware that a machine cannot be “empathic” in the strict sense. By this term we meant the ability of the VAs to vary the “tone of the answer” (e.g., happy, sad, reassuring, worried) based on the potential feelings (positive or negative) associated with the question asked by the user.

Based on our findings, VAs are far from being able to replace a visit to a male sexual health specialist. In any case, like any internet-based tool, VAs should simply have the aim of directing the patient to a competent doctor and reducing the patient’s doubts and anxieties about their state of health by providing scientifically correct, complete, clear, and empathic information. We have shown that significant efforts are still needed to make VAs reliable tools in this regard.

There are no studies in the literature specifically investigating VAs in the context of male sexual health; therefore, it is impossible to make direct comparisons with our results. However, some articles on the use of VAs in other clinical settings or areas of sexual health are available, allowing for indirect and more general comparisons. Hong et al. [46] compared the ability of Alexa, Siri, Google Assistant, and Cortana to understand and accurately respond to questions about cancer screening. There were clear differences among them and, almost unanimously, their verbal responses were either unavailable or inaccurate. Miner et al. [10] reported that Siri, Google Now, Cortana, and S Voice responded inconsistently and incompletely to simple questions about mental health, interpersonal violence, and physical health. Alagha et al. [9] evaluated the quality of responses regarding vaccines from Alexa, Siri, and Google Assistant. The authors found a high variability of results between the VAs. Additionally, Alexa underperformed compared with other VAs; this finding is consistent with our results. Wilson et al. [47] compared sexual health advice provided by Siri, Google Assistant, and a laptop-based Google search. This study also included a minority of questions about male sexual health. The authors found that the Google search performed much better than Siri and Google Assistant; in addition, they reported that Google Assistant performed better than Siri. The latter finding is inconsistent with our results. The authors concluded that people are able to find quality sexual health advice when searching online, but this is less likely if they use VAs, especially Siri.

### 4.2. Strengths and Limitations

To the best of our knowledge, this is the first study designed to specifically evaluate the ability of VAs to provide information on male sexual health; therefore, it could pave the way for a new line of research.

However, our results should be read considering several limitations. The most obvious weakness of our study is the lack of patient involvement. More specifically, the pool of questions was identified by the authors; no authoritative source of patient FAQs was found after an extensive online search. Moreover, the understandability of the responses for a patient was evaluated by the authors. Other limitations concern the device selection. Only one smart speaker for each VA was tested in the study; however, it is reasonable to assume that the answers do not vary between different smart speakers using the same VA since there is a shared technology behind it. Moreover, no device with a screen (smartphones or smart speakers) was tested, but this was decided to make the responses more easily comparable; in fact, devices with a screen often return answers that are partially or totally textual. The questions and answers were tested and evaluated in English; therefore, our results are not generalizable to other languages. Likewise, the study was conducted in Italy, and it is not possible to know if the questions formulated in another country would have received the same answers from the VAs. Furthermore, it is likely to hypothesize that responses change dynamically as search engine results change and cookie collection. Finally, the reliability of the VAs was evaluated for only a few of the many aspects of male sexual health with the selected FAQs.

### 4.3. Future Perspectives

VAs have a high potential in being used to search for information on male sexual health. In the near future, it is reasonable to hypothesize their growing diffusion and their increasing use for this purpose. A conscious and conscientious use of patients will be necessary to obtain good quality information from VAs; therefore, it would be desirable to find ways to educate patients on their use. A significant effort of developers would also be essential to improve VAs’ ability to recognize questions and the quality of answers. The selection of authoritative and up-to-date sources of information and the involvement of health professionals are certainly two key points of this improvement process. The incorporation of artificial intelligence (AI) in the technology of VAs to allow them to learn with experience and progressively improve is certainly a fascinating prospect for the future. While empathy is a critical aspect of doctor–patient communication, VAs generally provide “humanized but not human” responses, so special effort should be made to improve this issue. In parallel, further research should be conducted to evaluate the reliability of VAs in the male sexual health setting. Future studies should directly involve patients in selecting and formulating questions, as well as assessing the understandability of responses. Moreover, they should include comparison of different devices for each VA, questions in several languages and formulated in distinct countries, and exploration of other domains of male sexual function.

In the meantime, while technology and research on VAs continue to advance, a simple answer of “I am not adequate to help you, contact your doctor” to complex medical questions would certainly be desirable.

## 5. Conclusions

VAs are innovative but already widespread devices that can be used to search for information about male sexual health. The question recognition rate is quite high; however, the quality of the answers is still intermediate-low, and the language used lacks empathy. Siri seems superior to Alexa in both question recognition and response quality, while no clear difference emerges between Google Assistant and other VAs. Male infertility appears to be the sexual dysfunction best addressed by VAs among those investigated. The potential of VAs is huge, but a significant improvement in their technology is still needed to make them a reliable tool to search for information on sexual health. Further well-designed studies with direct patient involvement are desirable to elucidate the role of VAs in modern male sexual medicine.

## Figures and Tables

**Table 1:** Recognition of questions by VAs.

VA	Condition	Recognition of Question		
		Yes n (%)	No n (%)	Total n (%)
Siri	ED	28 (93.3)	2 (6.7)	30 (100)
	PE	24 (80.0)	6 (20.0)	30 (100)
	PD	20 (66.7)	10 (33.3)	30 (100)
	Male Infertility	30 (100)	0 (0.0)	30 (100)
	Other	23 (76.7)	7 (23.3)	30 (100)
	**Total**	**125 (83.3)**	**25 (16.7)**	**150 (100)**
Alexa	ED	28 (93.3)	2 (6.7)	30 (100)
	PE	12 (40.0)	18 (60.0)	30 (100)
	PD	10 (33.3)	20 (66.7)	30 (100)
	Male Infertility	28 (93.3)	2 (6.7)	30 (100)
	Other	18 (60.0)	12 (40.0)	30 (100)
	**Total**	**96 (64.0)**	**54 (36.0)**	**150 (100)**
Google Assistant	ED	28 (93.3)	2 (6.7)	30 (100)
	PE	18 (60.0)	12 (40.0)	30 (100)
	PD	15 (50.0)	15 (50.0)	30 (100)
	Male Infertility	29 (96.7)	1 (3.3)	30 (100)
	Other	21 (70.0)	9 (30.0)	30 (100)
	**Total**	**111 (74.0)**	**39 (26.0)**	**150 (100)**

VA: Voice Assistant; ED: Erectile Dysfunction; PE: Premature Ejaculation; PD: Peyronie’s Disease.

## Data Availability

Database available upon request.

## Supplementary Materials

The following supporting information can be downloaded at: https://www.mdpi.com/article/10.3390/ijerph20032612/s1, Table S1: Questions administered to VAs; Table S2. Quality and characteristics of the answers formulated by VAs; Table S3: Post hoc analysis: Comparison between VAs.
