# Supplementary material for: Voice Assistants as Consultants for Male Patients with Sexual Dysfunction: A Reliable Option?
Source: Int J Environ Res Public Health. 2023 Feb 1;20(3):2612. doi: 10.3390/ijerph20032612 (PMC9914936; doi:10.3390/ijerph20032612)
Supplement: Supplementary file 1 [file ijerph-20-02612-s001.zip › ijerph-2155781-supplementary.pdf]

Table S1. Questions administered to VAs

ED
What is impotence?
At what age can a man suffer from impotence?
How frequent is impotence?
Why does a man become impotent?
Can prostate problems cause impotence?
Does smoking and alcohol cause impotence?
I don't want to have sex anymore, what happens?
Who treats Impotence?
How do I know if I have impotence?
How is impotence treated?
Are there natural remedies for impotence?
Where can I buy impotence drugs?
How are impotence drugs taken?
What can i do if impotence drugs don't work?
What is a penile prosthesis?
**PE**
How long should a sexual intercourse last?
How long does it take a man to come normally?
When is ejaculation premature? What is definition of..
Is it normal to come immediately when you are young?
Why am I coming so early?
Could it be the anxiety that makes me come too early?
Who treats premature ejaculation?
How do I know if I have premature ejaculation?
How can I last longer during a sexual intercourse?
How is premature ejaculation treated?
Is premature ejaculation treatable?
Are there natural remedies for premature ejaculation?
Where can I buy drugs for premature ejaculation?
Does alcohol help not come too early?
Can I use an anesthetic for premature ejaculation?
**PD**
Why do I have a curved penis?
Why does my penis hurt when I have an erection?
Why is the penis bending?
My penis seems smaller to me, what happens?
What is Peyronie's disease?
I can feel a lump on my penis, what can it be?
Will my penis go straight?
Who treats a curved penis?
How is Peyronie's disease treated?
Can Peyronie's disease be cured?
Does the surgery for curved penis work?
Will my penis go straight back after surgery?
Will my penis go back as long as before after the operation?
Are there natural remedies for Peyronie's disease?
What alternatives are there to surgery?
**Male infertility**

What is infertility?
How does a man become sterile?
What are the causes of male infertility?
What is varicocele?
Can varicoceles cause Infertility?
Up to what age can a man have children?
Why can't I have children with my wife?
How do I know if I'm fertile?
What is a spermiogram?
How is a spermiogram performed?
Who treats male infertility?
How is male infertility treated?
Are there natural remedies for male infertility?
What is assisted reproduction?
When is it necessary to do assisted reproduction?
**Other**
How long is a normal penis?
How do I stretch my penis?
How do you make a woman come?
Where is G-spot?
Too much sex hurts?
How do I know if I'm a fetishist?
I'm afraid that I am a pedophile. What can I do?
What birth control methods are effective for not having children?
Too much masturbation hurts?
How can I avoid sexually transmistted diseases?
I feel a lump in my testicle, what should I do?
How do I know if I'm gay?
What is phimosis?
My testicle hurts, what can I do?
What is a condom for?

VA: Voice Assistant; ED: Erectile Dysfunction; PE: Premature Ejaculation; PD: Peyronie’s Disease.

Table S2. Quality and characteristics of the answers formulated by VAs.

Condition	Domain	VA	Mean	SD	95% CI *Lower Bound*	95% CI *Upper Bound*	Quality	P-value
ED	Targeted response to the problem	**Siri**	**5.40**	**4.073**	**3.88**	6.92	Intermediate	0.427
		Alexa	4.07	4.110	2.53	5.60	Intermediate	
		Google Assistant	4.90	3.745	3.50	6.30	Intermediate	
		Total	***4.79***	***3.973***	***3.96***	***5.62***	***Intermediate***	
	Scientific correctness of the answer	Siri	4.83	3.405	3.56	6.10	Intermediate	0.912
		Alexa	4.43	4.014	2.93	5.93	Intermediate	
		Google Assistant	4.67	3.417	3.39	5.94	Intermediate	
		Total	***4.64***	***3.586***	***3.89***	***5.40***	***Intermediate***	
	Completeness of the answer	Siri	4.60	3.692	3.22	5.98	Intermediate	0.811
		Alexa	3.97	4.064	2.45	5.48	Intermediate	
		Google Assistant	4.30	3.573	2.97	5.63	Intermediate	
		Total	***4.29***	***3.748***	***3.50***	***5.07***	***Intermediate***	
	Understandability of the response for a patient	Siri	5.27	3.552	3.94	6.59	Intermediate	0.558
		Alexa	4.33	3.960	2.85	5.81	Intermediate	
		Google Assistant	4.43	3.461	3.14	5.73	Intermediate	
		Total	***4.68***	***3.647***	***3.91***	***5.44***	***Intermediate***	
	Use of empathic language	Siri	2.13	2.156	0.98	3.27	Low	0.234
		Alexa	1.50	1.358	0.99	2.01	Low	
		Google Assistant	2.30	1.964	1.46	3.15	Low	
		Total	***1.91***	***1.788***	***1.48***	***2.34***	***Low***	
	Authoritativeness of the scientific source cited	Siri	3.47	3.481	2.17	4.77	Intermediate	**0.001**
		Alexa	0.67	1.204	0.16	1.18	Low	
		Google Assistant	2.41	2.653	1.40	3.42	Low	
		Total	***2.29***	***2.895***	***1.66***	***2.92***	***Low***	
	Referral to other appropriate sources of help	Siri	2.73	3.005	1.61	3.86	Low	**<0.0001**
		Alexa	0.13	.507	-0.06	0.32	Low	
		Google Assistant	1.69	2.494	0.74	2.64	Low	
		Total	***1.52***	***2.491***	***0.99***	***2.04***	***Low***	
PE	Targeted response to the problem	Siri	4.90	3.881	3.45	6.35	Intermediate	**<0.0001**
		Alexa	1.13	2.675	0.13	2.13	Low	
		Google Assistant	3.07	3.562	1.74	4.40	Intermediate	
		Total	***3.03***	***3.710***	***2.26***	***3.81***	***Intermediate***	
	Scientific correctness of the answer	Siri	3.47	2.813	2.42	4.52	Intermediate	**0.004**
		Alexa	1.10	2.537	0.15	2.05	Low	
		Google Assistant	2.30	2.693	1.29	3.31	Low	
		Total	***2.29***	***2.825***	***1.70***	***2.88***	***Low***	
	Completeness of the answer	Siri	4.27	3.383	3.00	5.53	Intermediate	**0.002**
		Alexa	1.20	2.858	0.13	2.27	Low	
		Google Assistant	2.73	3.290	1.50	3.96	Low	

		Total	***2.73***	***3.391***	***2.02***	***3.44***	***Low***	
	Understandability of the response for a patient	Siri	4.53	3.501	3.23	5.84	Intermediate	**<0.0001**
		Alexa	0.93	2.463	0.01	1.85	Low	
		Google Assistant	2.47	2.968	1.36	3.57	Low	
		Total	***2.64***	***3.323***	***1.95***	***3.34***	***Low***	
	Use of empathic language	Siri	0.43	0.514	0.13	0.73	Low	0.791
		Alexa	0.67	1.845	-0.02	1.36	Low	
		Google Assistant	0.82	1.842	0.00	1.63	Low	
		Total	***0.67***	***1.639***	***0.26***	***1.07***	***Low***	
	Authoritativeness of the scientific source cited	Siri	2.60	2.594	1.63	3.57	Low	**0.001**
		Alexa	0.47	0.973	0.10	0.83	Low	
		Google Assistant	1.90	2.455	0.96	2.83	Low	
		Total	***1.65***	***2.292***	***1.17***	***2.13***	***Low***	
	Referral to other appropriate sources of help	Siri	1.80	1.972	1.06	2.54	Low	**<0.0001**
		Alexa	0.07	0.254	-0.03	0.16	Low	
		Google Assistant	1.07	1.668	0.43	1.70	Low	
		Total	***0.98***	***1.644***	***0.63***	***1.32***	***Low***	
**PD**	Targeted response to the problem	Siri	3.97	4.064	2.45	5.48	Intermediate	0.098
		Alexa	1.90	3.438	0.62	3.18	Low	
		Google Assistant	2.67	3.585	1.33	4.01	Low	
		Total	***2.84***	***3.762***	***2.06***	***3.63***	***Low***	
	Scientific correctness of the answer	Siri	3.60	3.587	2.26	4.94	Intermediate	0.334
		Alexa	2.20	3.624	0.85	3.55	Low	
		Google Assistant	2.90	3.708	1.52	4.28	Low	
		Total	***2.90***	***3.644***	***2.14***	***3.66***	***Low***	
	Completeness of the answer	Siri	3.23	3.441	1.95	4.52	Intermediate	0.281
		Alexa	1.80	3.458	0.51	3.09	Low	
		Google Assistant	2.53	3.481	1.23	3.83	Low	
		Total	***2.52***	***3.471***	***1.80***	***3.25***	***Low***	
	Understandability of the response for a patient	Siri	3.53	3.461	2.24	4.83	Intermediate	0.156
		Alexa	1.83	3.217	0.63	3.03	Low	
		Google Assistant	2.63	3.459	1.34	3.92	Low	
		Total	***2.67***	***3.415***	***1.95***	***3.38***	***Low***	
	Use of empathic language	Siri	0.00	0.000	0.00	0.00	Low	0.211
		Alexa	0.67	1.295	0.18	1.15	Low	
		Google Assistant	0.55	1.335	-0.05	1.14	Low	
		Total	***0.48***	***1.180***	***0.19***	***0.77***	***Low***	
	Authoritativeness of the scientific source cited	Siri	3.00	3.173	1.82	4.18	Low	**0.001**
		Alexa	0.53	0.860	0.21	0.85	Low	
		Google Assistant	1.83	2.692	0.83	2.84	Low	
		Total	***1.79***	***2.629***	***1.24***	***2.34***	***Low***	
	Referral to other	Siri	1.93	3.005	0.81	3.06	Low	**0.004**
		Alexa	0.00	0.000	0.00	0.00	Low	

	appropriate sources of help	Google Assistant	1.23	2.329	0.36	2.10	Low	
		Total	***1.06***	***2.314***	***0.57***	***1.54***	***Low***	
**Male Infertility**	Targeted response to the problem	Siri	8.20	1.400	7.68	8.72	High	0.054
		Alexa	6.63	3.316	5.39	7.87	Intermediate	
		Google Assistant	7.13	2.474	6.21	8.06	High	
		Total	***7.32***	***2.578***	***6.78***	***7.86***	***High***	
	Scientific correctness of the answer	Siri	5.63	1.608	5.03	6.23	Intermediate	0.811
		Alexa	6.07	3.581	4.73	7.40	Intermediate	
		Google Assistant	6.00	2.804	4.95	7.05	Intermediate	
		Total	***5.90***	***2.760***	***5.32***	***6.48***	***Intermediate***	
	Completeness of the answer	Siri	5.07	1.760	4.41	5.72	Intermediate	0.950
		Alexa	5.10	3.241	3.89	6.31	Intermediate	
		Google Assistant	5.27	2.545	4.32	6.22	Intermediate	
		Total	***5.14***	***2.560***	***4.61***	***5.68***	***Intermediate***	
	Understandability of the response for a patient	Siri	5.93	1.363	5.42	6.44	Intermediate	0.243
		Alexa	4.97	3.253	3.75	6.18	Intermediate	
		Google Assistant	5.00	2.533	4.05	5.95	Intermediate	
		Total	***5.30***	***2.519***	***4.77***	***5.83***	***Intermediate***	
	Use of empathic language	Siri	3.00	0.000	3.00	3.00	Low	0.470
		Alexa	1.67	1.493	1.11	2.22	Low	
		Google Assistant	1.88	1.586	1.03	2.72	Low	
		Total	***1.79***	***1.501***	***1.36***	***2.23***	***Low***	
	Authoritativeness of the scientific source cited	Siri	3.93	2.212	3.11	4.76	Intermediate	**<0.0001**
		Alexa	1.43	1.716	0.79	2.07	Low	
		Google Assistant	2.67	2.023	1.91	3.42	Low	
		Total	***2.68***	***2.223***	***2.21***	***3.14***	***Low***	
	Referral to other appropriate sources of help	Siri	3.20	1.400	2.68	3.72	Intermediate	**<0.0001**
		Alexa	0.00	0.000	0.00	0.00	Low	
		Google Assistant	2.07	1.751	1.40	2.74	Low	
		Total	***1.75***	***1.848***	***1.36***	***2.14***	***Low***	
**Other**	Targeted response to the problem	Siri	6.20	3.899	4.74	7.66	Intermediate	**0.020**
		Alexa	3.37	3.970	1.88	4.85	Intermediate	
		Google Assistant	4.40	3.775	2.99	5.81	Intermediate	
		Total	***4.66***	***4.014***	***3.81***	***5.50***	***Intermediate***	
	Scientific correctness of the answer	Siri	4.00	3.523	2.68	5.32	Intermediate	0.566
		Alexa	3.03	3.634	1.68	4.39	Intermediate	
		Google Assistant	3.63	3.429	2.35	4.91	Intermediate	
		Total	***3.56***	***3.513***	***2.82***	***4.29***	***Intermediate***	
	Completeness of the answer	Siri	5.07	3.453	3.78	6.36	Intermediate	0.070
		Alexa	2.90	3.661	1.53	4.27	Low	
		Google Assistant	3.93	3.648	2.57	5.30	Intermediate	
		Total	***3.97***	***3.658***	***3.20***	***4.73***	***Intermediate***	

Understandability of the response for a patient	Siri	5.13	3.170	3.95	6.32	Intermediate	0.118
	Alexa	3.30	3.678	1.93	4.67	Intermediate	
	Google Assistant	4.00	3.404	2.73	5.27	Intermediate	
	Total	***4.14***	***3.469***	***3.42***	***4.87***	***Intermediate***	
Use of empathic language	Siri	1.00	1.907	-0.21	2.21	Low	0.288
	Alexa	0.33	0.922	-0.01	0.68	Low	
	Google Assistant	0.52	1.123	0.01	1.04	Low	
	Total	***0.52***	***1.229***	***0.21***	***0.83***	***Low***	
Authoritativeness of the scientific source cited	Siri	3.20	2.882	2.12	4.28	Intermediate	**<0.0001**
	Alexa	0.60	1.070	0.20	1.00	Low	
	Google Assistant	1.87	2.432	0.96	2.77	Low	
	Total	***1.89***	***2.479***	***1.37***	***2.41***	***Low***	
Referral to other appropriate sources of help	Siri	0.87	0.819	0.56	1.17	Low	0.535
	Alexa	0.67	1.882	-0.04	1.37	Low	
	Google Assistant	0.50	0.777	0.21	0.79	Low	
	Total	***0.68***	***1.262***	***0.41***	***0.94***	***Low***	

VA: Voice Assistant; SD: Standard Deviation; CI: Confidence Interval; ED: Erectile Dysfunction; PE: Premature Ejaculation; PD: Peyronie's Disease.

Arbitrary mean scores of 0-3 were associated with low quality, > 3 but < 7 with intermediate quality, and 7-10 with high quality.

Statistically significant p-values were marked in bold.

Table S3. Post hoc analysis: Comparison between VAs

**Condition**	**Domain (Dependent Variable)**	**VA 1**	**VA 2**	**Mean Difference**	**Standard Error**	**P-value**
**ED**	Targeted response to the problem	Siri	Alexa	1.333	1.028	0.594
			Google Assistant	0.500	1.028	1.000
		Alexa	Siri	-1.333	1.028	0.594
			Google Assistant	-0.833	1.028	1.000
		Google Assistant	Siri	-0.500	1.028	1.000
			Alexa	0.833	1.028	1.000
	Scientific correctness of the answer	Siri	Alexa	0.400	0.936	1.000
			Google Assistant	0.167	0.936	1.000
		Alexa	Siri	-0.400	0.936	1.000
			Google Assistant	-0.233	0.936	1.000
		Google Assistant	Siri	-0.167	0.936	1.000
			Alexa	0.233	0.936	1.000
	Completeness of the answer	Siri	Alexa	0.633	0.977	1.000
			Google Assistant	0.300	0.977	1.000
		Alexa	Siri	-0.633	0.977	1.000
			Google Assistant	-0.333	0.977	1.000
		Google Assistant	Siri	-0.300	0.977	1.000
			Alexa	0.333	0.977	1.000
	Understandability of the response for a patient	Siri	Alexa	0.933	0.946	0.980
			Google Assistant	0.833	0.946	1.000
		Alexa	Siri	-0.933	0.946	0.980
			Google Assistant	-0.100	0.946	1.000
		Google Assistant	Siri	-0.833	0.946	1.000
			Alexa	0.100	0.946	1.000
	Use of empathic language	Siri	Alexa	0.625	0.550	0.779
			Google Assistant	-0.179	0.578	1.000
		Alexa	Siri	-0.625	0.550	0.779
			Google Assistant	-0.804	0.492	0.321
		Google Assistant	Siri	0.179	0.578	1.000
			Alexa	0.804	0.492	0.321
	Authoritativeness of the scientific source cited	Siri	Alexa	2.800	0.739	**0.001**
			Google Assistant	1.053	0.702	0.413
		Alexa	Siri	-2.800	0.739	**0.001**
			Google Assistant	-1.747	0.744	0.064
		Google Assistant	Siri	-1.053	0.702	0.413
			Alexa	1.747	0.744	0.064
		Siri	Alexa	2.600	0.586	**<0.0001**

	Referral to other appropriate sources of help		Google Assistant	1.044	0.591	0.243
		Alexa	Siri	-2.600	0.586	**<0.0001**
			Google Assistant	-1.556	0.591	**0.030**
		Google Assistant	Siri	-1.044	0.591	0.243
			Alexa	1.556	0.591	**0.030**
**PE**	Targeted response to the problem	Siri	Alexa	3.767	0.881	**<0.0001**
			Google Assistant	1.833	0.881	0.121
		Alexa	Siri	-3.767	0.881	**<0.0001**
			Google Assistant	-1.933	0.881	0.092
		Google Assistant	Siri	-1.833	0.881	0.121
			Alexa	1.933	0.881	0.092
	Scientific correctness of the answer	Siri	Alexa	2.367	0.693	**0.003**
			Google Assistant	1.167	0.693	0.287
		Alexa	Siri	-2.367	0.693	**0.003**
			Google Assistant	-1.200	0.693	0.260
		Google Assistant	Siri	-1.167	0.693	0.287
			Alexa	1.200	0.693	0.260
	Completeness of the answer	Siri	Alexa	3.067	0.822	**0.001**
			Google Assistant	1.533	0.822	0.197
		Alexa	Siri	-3.067	0.822	**0.001**
			Google Assistant	-1.533	0.822	0.197
		Google Assistant	Siri	-1.533	0.822	0.197
			Alexa	1.533	0.822	0.197
	Understandability of the response for a patient	Siri	Alexa	3.600	0.776	**<0.0001**
			Google Assistant	2.067	0.776	**0.028**
		Alexa	Siri	-3.600	0.776	**<0.0001**
			Google Assistant	-1.533	0.776	0.154
		Google Assistant	Siri	-2.067	0.776	**0.028**
			Alexa	1.533	0.776	0.154
	Use of empathic language	Siri	Alexa	-0.238	0.537	1.000
			Google Assistant	-0.390	0.567	1.000
		Alexa	Siri	0.238	0.537	1.000
			Google Assistant	-0.152	0.466	1.000
		Google Assistant	Siri	0.390	0.567	1.000
			Alexa	0.152	0.466	1.000
	Authoritativeness of the scientific source cited	Siri	Alexa	2.133	0.551	**0.001**
			Google Assistant	0.703	0.556	0.626
		Alexa	Siri	-2.133	0.551	**0.001**
			Google Assistant	-1.430	0.556	**0.035**

		Google Assistant	Siri	-0.703	0.556	0.626
			Alexa	1.430	0.556	**0.035**
	Referral to other appropriate sources of help	Siri	Alexa	1.733	0.386	**<0.0001**
			Google Assistant	0.731	0.390	0.192
		Alexa	Siri	-1.733	0.386	**<0.0001**
			Google Assistant	-1.002	0.390	**0.035**
		Google Assistant	Siri	-0.731	0.390	0.192
			Alexa	1.002	0.390	**0.035**
**PD**	Targeted response to the problem	Siri	Alexa	2.067	0.957	0.100
			Google Assistant	1.300	0.957	0.533
		Alexa	Siri	-2.067	0.957	0.100
			Google Assistant	-0.767	0.957	1.000
		Google Assistant	Siri	-1.300	0.957	0.533
			Alexa	0.767	0.957	1.000
	Scientific correctness of the answer	Siri	Alexa	1.400	0.940	0.420
			Google Assistant	0.700	0.940	1.000
		Alexa	Siri	-1.400	0.940	0.420
			Google Assistant	-0.700	0.940	1.000
		Google Assistant	Siri	-0.700	0.940	1.000
			Alexa	0.700	0.940	1.000
	Completeness of the answer	Siri	Alexa	1.433	0.893	0.337
			Google Assistant	0.700	0.893	1.000
		Alexa	Siri	-1.433	0.893	0.337
			Google Assistant	-0.733	0.893	1.000
		Google Assistant	Siri	-0.700	0.893	1.000
			Alexa	0.733	0.893	1.000
	Understandability of the response for a patient	Siri	Alexa	1.700	0.873	0.164
			Google Assistant	0.900	0.873	0.916
		Alexa	Siri	-1.700	0.873	0.164
			Google Assistant	-0.800	0.873	1.000
		Google Assistant	Siri	-0.900	0.873	0.916
			Alexa	0.800	0.873	1.000
	Use of empathic language	Siri	Alexa	-0.667	0.378	0.249
			Google Assistant	-0.545	0.400	0.532
		Alexa	Siri	0.667	0.378	0.249
			Google Assistant	0.121	0.328	1.000
		Google Assistant	Siri	0.545	0.400	0.532
			Alexa	-0.121	0.328	1.000
	Authoritativeness of the scientific source cited	Siri	Alexa	2.467	0.633	**0.001**
			Google Assistant	1.167	0.633	0.207

		Alexa	Siri	-2.467	0.633	**0.001**
			Google Assistant	-1.300	0.633	0.129
		Google Assistant	Siri	-1.167	0.633	0.207
			Alexa	1.300	0.633	0.129
	Referral to other appropriate sources of help	Siri	Alexa	1.933	0.567	**0.003**
			Google Assistant	0.700	0.567	0.660
		Alexa	Siri	-1.933	0.567	**0.003**
			Google Assistant	-1.233	0.567	0.097
		Google Assistant	Siri	-0.700	0.567	0.660
			Alexa	1.233	0.567	0.097
**Male Infertility**	Targeted response to the problem	Siri	Alexa	1.567	0.651	0.055
			Google Assistant	1.067	0.651	0.315
		Alexa	Siri	-1.567	0.651	0.055
			Google Assistant	-0.500	0.651	1.000
		Google Assistant	Siri	-1.067	0.651	0.315
			Alexa	0.500	0.651	1.000
	Scientific correctness of the answer	Siri	Alexa	-0.433	0.719	1.000
			Google Assistant	-0.367	0.719	1.000
		Alexa	Siri	0.433	0.719	1.000
			Google Assistant	0.067	0.719	1.000
		Google Assistant	Siri	0.367	0.719	1.000
			Alexa	-0.067	0.719	1.000
	Completeness of the answer	Siri	Alexa	-0.033	0.668	1.000
			Google Assistant	-0.200	0.668	1.000
		Alexa	Siri	0.033	0.668	1.000
			Google Assistant	-0.167	0.668	1.000
		Google Assistant	Siri	0.200	0.668	1.000
			Alexa	0.167	0.668	1.000
	Understandability of the response for a patient	Siri	Alexa	0.967	0.647	0.417
			Google Assistant	0.933	0.647	0.459
		Alexa	Siri	-0.967	0.647	0.417
			Google Assistant	-0.033	0.647	1.000
		Google Assistant	Siri	-0.933	0.647	0.459
			Alexa	0.033	0.647	1.000
	Use of empathic language	Siri	Alexa	1.333	1.102	0.698
			Google Assistant	1.125	1.131	0.976
		Alexa	Siri	-1.333	1.102	0.698
			Google Assistant	-0.208	0.467	1.000
		Google Assistant	Siri	-1.125	1.131	0.976
			Alexa	0.208	0.467	1.000

	Authoritativeness of the scientific source cited	Siri	Alexa	2.500	0.515	**<0.0001**
			Google Assistant	1.267	0.515	**0.048**
		Alexa	Siri	-2.500	0.515	**<0.0001**
			Google Assistant	-1.233	0.515	0.056
		Google Assistant	Siri	-1.267	0.515	**0.048**
			Alexa	1.233	0.515	0.056
	Referral to other appropriate sources of help	Siri	Alexa	3.200	0.333	**<0.0001**
			Google Assistant	1.131	0.335	**0.003**
		Alexa	Siri	-3.200	0.333	**<0.0001**
			Google Assistant	-2.069	0.335	**<0.0001**
		Google Assistant	Siri	-1.131	0.335	**0.003**
			Alexa	2.069	0.335	**<0.0001**
**Other**	Targeted response to the problem	Siri	Alexa	2.833	1.002	**0.017**
			Google Assistant	1.800	1.002	0.228
		Alexa	Siri	-2.833	1.002	**0.017**
			Google Assistant	-1.033	1.002	0.916
		Google Assistant	Siri	-1.800	1.002	0.228
			Alexa	1.033	1.002	0.916
	Scientific correctness of the answer	Siri	Alexa	0.967	0.911	0.875
			Google Assistant	0.367	0.911	1.000
		Alexa	Siri	-0.967	0.911	0.875
			Google Assistant	-0.600	0.911	1.000
		Google Assistant	Siri	-0.367	0.911	1.000
			Alexa	0.600	0.911	1.000
	Completeness of the answer	Siri	Alexa	2.167	0.927	0.065
			Google Assistant	1.133	0.927	0.674
		Alexa	Siri	-2.167	0.927	0.065
			Google Assistant	-1.033	0.927	0.804
		Google Assistant	Siri	-1.133	0.927	0.674
			Alexa	1.033	0.927	0.804
	Understandability of the response for a patient	Siri	Alexa	1.833	0.884	0.123
			Google Assistant	1.133	0.884	0.610
		Alexa	Siri	-1.833	0.884	0.123
			Google Assistant	-0.700	0.884	1.000
		Google Assistant	Siri	-1.133	0.884	0.610
			Alexa	0.700	0.884	1.000
	Use of empathic language	Siri	Alexa	0.667	0.418	0.348
			Google Assistant	0.476	0.443	0.860
		Alexa	Siri	-0.667	0.418	0.348

			Google Assistant	-0.190	0.348	1.000
		Google Assistant	Siri	-0.476	0.443	0.860
			Alexa	0.190	0.348	1.000
	Authoritativeness of the scientific source cited	Siri	Alexa	2.600	0.584	**<0.0001**
			Google Assistant	1.333	0.584	0.075
		Alexa	Siri	-2.600	0.584	**<0.0001**
			Google Assistant	-1.267	0.584	0.099
		Google Assistant	Siri	-1.333	0.584	0.075
			Alexa	1.267	0.584	0.099
	Referral to other appropriate sources of help	Siri	Alexa	0.200	0.327	1.000
			Google Assistant	0.367	0.327	0.796
		Alexa	Siri	-0.200	0.327	1.000
			Google Assistant	0.167	0.327	1.000
		Google Assistant	Siri	-0.367	0.327	0.796
			Alexa	-0.167	0.327	1.000

VA: Voice Assistant; SD: Standard Deviation; CI: Confidence Interval; ED: Erectile Dysfunction; PE: Premature Ejaculation; PD: Peyronie's Disease.

Statistically significant p-values were marked in bold.
